# Polychlorinated Biphenyl Exposure and Neuropsychological Status among Older Residents of Upper Hudson River Communities

**DOI:** 10.1289/ehp.10432

**Published:** 2007-11-12

**Authors:** Edward F. Fitzgerald, Erin E. Belanger, Marta I. Gomez, Michael Cayo, Robert J. McCaffrey, Richard F. Seegal, Robert L. Jansing, Syni-an Hwang, Heraline E. Hicks

**Affiliations:** 1 Department of Epidemiology and Biostatistics, School of Public Health, University at Albany, State University of New York, Rensselaer, New York, USA; 2 Bureau of Environmental and Occupational Epidemiology, Center for Environmental Health, New York State Department of Health, Troy, New York, USA; 3 Department of Psychology, College of Liberal Arts and Sciences, University at Albany, State University of New York, Albany, New York, USA; 4 Division of Environmental Disease Prevention, Wadsworth Center, New York State Department of Health, Albany, New York, USA; 5 Division of Toxicology and Environmental Medicine, Agency for Toxic Substances and Disease Registry, Atlanta, Georgia, USA

**Keywords:** adult, affective symptoms, hazardous waste, neurobehavioral manifestations, neuropsychological tests, polychlorinated biphenyls

## Abstract

**Background:**

Polychlorinated biphenyls (PCBs) may accelerate the cognitive and motor dysfunction found in normal aging, but few studies have examined these outcomes and PCB exposure among older adults.

**Objective:**

We evaluated neuropsychological status and low-level PCB exposure among older adults living along contaminated portions of the upper Hudson River in New York.

**Methods:**

A total of 253 persons between 55 and 74 years of age were recruited and interviewed, and provided blood samples for congener-specific PCB analysis. Participants also underwent a neuropsychological battery consisting of 34 tests capable of detecting subtle deficits in cognition, motor function, affective state, and olfactory function.

**Results:**

After adjustment for potential confounders, the results indicated that an increase in serum total PCB concentration from 250 to 500 ppb (lipid basis) was associated with a 6.2% decrease in verbal learning, as measured by California Verbal Learning Test trial 1 score (*p* = 0.035), and with a 19.2% increase in depressive symptoms, as measured by the Beck Depression Inventory (*p* = 0.007).

**Conclusions:**

The results suggest that exposure to PCBs may be associated with some measures of memory and learning and depression among adults 55–74 years of age whose current body burdens are similar to those of the general population. Although the results are useful in delineating the neuropsychological effects of low-level exposure to PCBs, further studies of whether older men and women are a sensitive subpopulation are needed.

Considerable evidence links polychlorinated biphenyls (PCBs) to subtle neurodevelopmental deficits among neonates, infants, and children (Schantz et al. 2003). Relatively few studies, however, address neurotoxicity among adults exposed to PCBs (Faroon et al. 2002). The possibility that PCBs may interact with the neurodegenerative processes associated with aging underscores the need for more data on long-term neurologic effects of PCBs in adults (Seegal 2001). To date, only one study has examined these effects. In a study of aging Great Lakes fish consumers, Schantz et al. (2001) reported that PCB exposure was negatively associated with several measures of memory and learning, whereas other cognitive domains and motor function (Schantz et al. 1999) were unaffected. The present study assesses the neuropsychological status of older adults living along PCB-contaminated portions of the Hudson River in New York.

Over approximately 30 years, General Electric plants in Fort Edward and Hudson Falls, New York, used PCBs to manufacture electrical capacitors. Nearly 1 million pounds of PCBs were discharged from these facilities into the Hudson River, about 15% of the nationwide discharge at that time (Hetling et al. 1978). In a previous article (Fitzgerald et al. 2007), we reported that although some older long-term residents of Fort Edward and Hudson Falls ate fish from a contaminated portion of the Hudson River in the past, their current rates of consumption are low, apparently due to the fishing ban and advisories issued by state environmental and health agencies. After adjustment for age, body mass index (BMI), and cigarette smoking, the geometric mean serum total PCB concentration of Fort Edward and Hudson Falls residents was 3.07 ppb (wet weight) and 473 ppb (lipid basis), values that did not differ significantly from 3.23 ppb (wet weight) and 485 ppb (lipid basis) for residents of Glens Falls, a control community upriver from the PCB contamination. Despite low current consumption rates, serum PCB levels tended to increase with estimated cumulative lifetime exposure to PCBs from Hudson River fish consumption.

The current article extends the investigation by examining neuropsychological status in these populations to evaluate whether low-level PCB exposure is associated with nervous system function. This study builds on the work of Schantz et al. (1999, 2001) and extends the outcome assessment to include domains such as affective state and olfactory function that they did not investigate. Given the possible effects of PCBs on dopamine and serotonin (Morse et al. 1996; Seegal et al. 1991) and the impact of these neurotransmitters on cognition (Volkow et al. 1998), mood (Baldwin and Rudge 1995), and olfaction (Doty et al. 1992), it is hypothesized that serum PCB concentrations will positively associate with measures of depression and anxiety and negatively associate with learning, memory, and smell. In order for us to evaluate the neurotoxicity of specific congeners, the exposure assessment includes serum levels of individual PCB congeners as well as total PCBs.

## Materials and Methods

### Study population, recruitment, and interview

Participants were randomly selected from several sources, including an on-line telephone directory search and a digital database from *info*USA (Omaha, NE). Excluding persons who were not 55–74 years of age, there were 1,281 candidates in Fort Edward and Hudson Falls and 1,423 in Glens Falls. Participants were then screened by telephone for eligibility.

Overall, 1,125 (88%) candidates in the study area and 1,228 (86%) in the comparison area were able to be contacted. Of these persons, 85% agreed to be screened; 482 (50%) were eligible in the study area, 419 (41%) in the comparison area. People who had not lived in their respective area for at least 25 years (27%) were excluded. Persons with a history of stroke, severe head injury, signs of Parkinson or Alzheimer disease, or other significant cognitive or motor impairment (17%) were also excluded, as persons with severe cognitive disabilities would have difficulty completing the neuropsychological tests and interview. To avoid confounding with occupational PCB exposures, we also excluded anyone who had worked for 1 year or more at the capacitor plants or any other job that entailed PCB exposure (21%). Persons from Glens Falls were frequency matched to the Hudson Falls and Fort Edward residents on both age and sex.

Structured in-person interviews were conducted from 2000 to 2002; questions included sociodemographic characteristics, height, weight, residential, occupational, and dietary history, cigarette smoking, alcohol consumption, health conditions, medication use, hobbies, and other characteristics and behaviors. Occupational histories were reviewed by two industrial hygienists for the probability of occupational exposure to lead, mercury, solvents, or pesticides.

Among those who were eligible and invited to participate, the response rates were 38% in the study area and 41% in the comparison area. There were no differences in participation according to age or sex. After the data collection was completed, 53 participants were excluded because they failed to have blood drawn, the laboratory was unable to analyze the serum for PCBs, or their interview indicated a reason for exclusion not previously reported.

**Figure 1:**
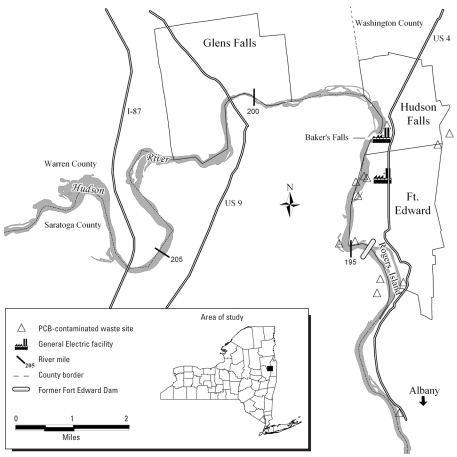
Map of the area of study: Hudson Falls, Fort Edward, and Glens Falls, New York.

Given their similarity in serum PCB concentrations, the study and comparison groups were combined. As a result, the total sample consisted of 253 persons 55–74 years of age. Sixty-seven men and 66 women were residents of Hudson Falls or Fort Edward, New York, and 60 men and 60 women were residents of Glens Falls (Figure 1). More detailed descriptions of the populations, recruitment, and interviews are given elsewhere (Fitzgerald et al. 2007).

### Serum PCB analysis

Fasting serum samples were analyzed by dual capillary gas chromatography with microelectron capture detection for the 30 congeners that typically constitute more than 95% of the total PCB residue in human serum (Humphrey et al. 2000); their sum was calculated as total PCB. The method detection limit was 0.02 ppb per congener; nondetectable concentrations were assigned a value of one-half the detection limit. However, data are only presented for individual congeners if 50% or more of the samples had a detectable concentration. To provide for comparability with other studies, cholesterol and triglycerides were assayed enzymatically for 245 persons and their PCB concentrations expressed on a lipid basis (Phillips et al. 1989), although Schisterman et al. (2005) recently have argued that this method of lipid adjustment may introduce bias. In addition, nine dioxin-like PCB congeners were measured in the serum of about 90% of the study participants, and PCB toxic equivalent quantities (TEQs) were calculated (Van den Berg et al. 2006). Twenty-two organochlorine pesticides were also measured, but most were nondetectable. The only major exceptions were dichlorodiphenyl trichlorethane (DDT) and its metabolite *p,p*-dichlorodiphenyl dichloroethene (DDE). Because the concentrations of DDE were on average 20 times greater than those for DDT, both compounds were summed and evaluated jointly. More detail on the chemical analysis, including quality assurance/quality control procedures, is reported elsewhere (Fitzgerald et al. 2007).

### Blood metals analysis

For blood lead analysis, whole blood was diluted 1:10 in a phosphate matrix modifier containing Triton X-100 and dilute nitric acid, and 12 μL was deposited into a graphite furnace atomic adsorption spectrometry instrument that incorporated longitudinal Zeeman background correction with a transversely heated graphite atomizer (Parsons and Slavin 1993). For the blood mercury analysis, an inductively coupled plasma–mass spectrometer was used (Palmer et al. 2006). The collection tubes were trace metal-free to avoid background contamination.

### Neuropsychological assessment

The neuropsychological tests were selected because they provide sensitive, clinically relevant measures of nervous system functions that are altered by aging and by damage to central dopamine processes. Not only have these tests been shown to be altered in studies of individuals occupationally exposed to PCBs (Singer 1988; Troster et al. 1991), but they also have been used in PCB research involving nonoccupationally exposed groups (Schantz et al. 1999, 2001), thereby facilitating a comparison of results between studies. In addition, olfactory function was assessed, as a decreased sense of smell is among the first signs of idiopathic diseases involving the depletion of brain dopamine (Doty et al. 1992). The New Adult Reading Test-Revised (NART-R) was used to assess intelligence (Nelson and O’Connell 1978), given that intellectual ability is a key determinant of performance on cognitive tests. The Test of Memory Malingering (TOMM) was used to discriminate between participants who were putting forth an adequate level of effort and those who were not (Tombaugh 1996).

Memory and learning was assessed by two different tests: the California Verbal Learning Test (CVLT) (Delis et al. 2000) and the Weschler Memory Scale (WMS) Form I-Russell’s Revision (Russell 1975). The CVLT test uses semantic associations as a strategy to learning words in a 16-item list. The WMS was used to assess immediate and delayed recall of verbal and visual material.

Executive functioning pertains to abstract reasoning, concept formation, and other higher-order cognitive abilities. It was assessed with four different tests: the Trail Making Test-Parts A and B, the Stroop Color–Word Test (SCWT), and the Wisconsin Card Sorting Test (WCST). The Trail Making Test-Parts A and B is a subtest of the Halstead-Reitan Battery (Reitan and Wolfson 1993) and assesses visual scanning and attention. The SCWT consists of three parts and assesses the participant’s ability to shift a perceptual set (Treverry et al. 1994). The WCST was used to assess concept formation and set shifting strategies.

The visual spatial domain pertains to the ability to interact with the environment visually and spatially. Two tests were used: the Digit Symbol Substitution Test (DSST), and the Block Design subtest (BDT) from the Wechsler Adult Intelligence Test-Revised (Wechsler 1981). The DSST was used to assess passive associative learning, visual speed, attention, and intense effort. The BDT was used to measure visuospatial organization.

Three tests were employed to assess motor function: the Static Motor Steadiness Test (SMST), the Grooved Pegboard Test (GPT), and the Finger Oscillation Test (FOT). The SMST assessed for tremorlike movements in the participants (Lezak et al. 2004). The GPT (Klove 1963) evaluated complex visuomotor coordination and visual–spatial orientation. The FOT is a subtest of the Halstead-Reitan Neuropsychological Battery and was used as a measure of motor speed and coordination (Reitan and Wolfson 1993).

Simple reaction time was measured after the participant was told to respond to the appearance of a visual stimulus after an auditory warning.

Depression and anxiety were assessed using the Beck Depression Inventory (BDI) and the State-Trait Anxiety Inventory (STAI) respectively. The BDI is a 21-item self-rated scale that measures the graded severity of certain symptoms of depression (Beck et al. 1961). The STAI was used to measure both state anxiety and trait anxiety. The instrument consisted of two scales of 20 items each (Speilberger et al. 1970); one measured state anxiety and the other measured trait.

Finally, olfactory function was measured using the Smell Identification Test (SIT), a 40-item scratch and sniff multiple-choice test (Doty 1983) that is widely used to assess olfactory functioning both clinically and experimentally.

### Statistical analyses

Multiple linear regression analysis was used to test for association between PCB exposure and neuropsychological test scores after controlling for significant background variables that could potentially confound such associations. Following the method of Schantz et al. (1999, 2001), potential confounding variables were first screened for each neuropsychological test in a bivariate analysis. Candidate variables included age, sex, education, income, NART-R score, BMI, marital status, cigarette smoking, alcohol consumption, health conditions, medication use, employment status, physical activity level, hours of sleep, blood lead and mercury concentrations, serum DDT + DDE level, and occupational or hobby exposure to lead, mercury, solvents, or pesticides. Regarding smoking, participants reported the number of cigarettes smoked over the past year and the past 10 years, which in turn was then converted to packs smoked (nonsmokers were assigned zero). In addition to being analyzed as outcome variables, depression and anxiety were also considered as potential confounders in the analysis of the cognitive and motor tests, as they may affect performance in those domains. Variables that were significant in the bivariate analysis at *p* < 0.20 and considered biologically plausible by our consulting psychologist (R.J.M.) were regressed on the neuropsychological test scores using stepwise procedures to add (*p* < 0.10) or remove (*p* > 0.10) the variables one at a time. New regression models were then created with the addition of serum PCB concentrations to estimate their associations with the neuropsychological test scores after adjustment for potential confounders.

Serum PCB and DDE concentrations were expressed on a lipid basis and log transformed; blood lead and mercury concentrations were log transformed. When the distribution of scores on a given neuropsychological test was skewed, the scores were also log transformed. For a few tests, the log transformations failed to achieve normality, so the scores were dichotomized at the median and logistic regression performed. For some neuropsychological tests, serum PCB levels were divided into quartiles and a test for linear trend conducted, coding the categories as 1 through 4. The data were also stratified by age, sex, and income to assess effect modification.

## Results

Table 1 summarizes the background characteristics of the 253 study participants. Reflecting the study design, age ranged from 55–74 years with a mean of 63.9, and 50% were men. Nearly 60% had some college education. Twenty percent were current cigarette smokers and 27% were past smokers (data not shown). Ninety-seven percent of all participants were white, and 99% were non-Hispanic (data not shown). According to the industrial hygienists, fewer than 5% were occupationally exposed to mercury or pesticides (data not shown). The mean serum total PCB concentration was 3.6 ppb (wet weight) or 537 ppb (lipid basis). The nine dioxin-like PCB congeners were measured in the serum of 232 participants, and the mean TEQ concentration was 34.2 ppt (lipid basis). The means were 4.0 ppb (wet weight) or 738 ppb (lipid basis) for serum DDT + DDE, 3.1 μg/dL for blood lead and 0.30 μg/dL for blood mercury.

Table 2 lists background variables included in the final regression models for each neuropsychological test by domain. For many of the cognitive tests, performance was lower among men, decreased with age, and increased with intellectual function, education, and income. Reaction time decreased with age, and was lower among smokers, arthritics, and persons who reported fewer hours of sleep per week and who did not take nonsteroidal anti-inflammatory drugs (NSAIDs). Older persons and those with arthritis also performed less well on the pegboard test of motor function. BDI scores were higher among persons who reported using antidepressants and gout medications, whereas state anxiety and trait anxiety were higher among those who used sex hormones. Olfactory function was lower among older persons and those who smoked. Scores on the TOMM were within normal limits, indicating that the level of effort of all of the participants was adequate.

Table 3 displays the final multivariate models for the neuropsychological tests with log-transformed serum total PCB concentration after adjusting for the covariates in Table 2. In the memory and learning domain, performance on the CVLT trial 1 score, declined as log serum total PCB concentration, increased (β = −0.576 per unit change in log-adjusted lipid basis serum PCB, *p* = 0.035). Specifically, an increase in serum total PCB concentration from 250 to 500 ppb (lipid basis) was associated with a 6.2% mean decrease in performance. The BDI increased with log serum total PCB concentration (β = 1.189, *p* = 0.007). That is, as serum total PCB concentration increased from 250 to 500 ppb (lipid basis), depressive symptoms increased on average by 19.2%. Only two participants, however, had a score on the BDI that indicated moderate to severe depression; the results were unchanged when the 15 participants who reported taking antidepressants were excluded. The only other significant association was for the WMS test of visual immediate recall (β= 1.016, *p* = 0.012). In this case, performance improved by 8.9% as serum total PCB concentration increased from 250 to 500 ppb (lipid basis).

To further examine the associations between PCB exposure and the CVLT trial 1 score and the BDI, we divided the study population into quartiles of serum total PCB concentration and compared their adjusted mean test scores (Table 4). The results indicated that the deficits in the CVLT trial 1 score were limited to the highest PCB quartile, whereas scores on the BDI rose in both the third and fourth quartiles. Additional analyses by age and sex (not shown) revealed that the association between serum total PCB concentration and the CVLT trial 1 score was significant among persons 55–64 years of age (β = −0.872 per unit change in log-adjusted lipid basis serum PCB, *p* = 0.038) but not significant among those 65–74 years of age (β = −0.406, *p* = 0.311), and similarly significant among men (β = −0.958, *p* = 0.021) but not women (β = −0.372, *p* = 0.314). To further evaluate these differences, age was categorized into three groups and analyzed jointly with sex. The results indicated that the PCB–CVLT trial 1 score association was strongest among men 55–60 years of age (β = 1.797, *p* = 0.006). Effect modification by income was also observed for the CVLT, trial 1 score, with a significant association with serum PCB level only apparent for those with total family incomes of < $45,000 (β = −0.893, *p* = 0.017 versus β = −0.384, *p* = 0.403 for those with incomes of ≥$45,000).

The association between serum total PCB concentration and the BDI was also significant among persons 55–64 years of age (β = 2.934, *p* = 0.002) and not for persons age 65–74 years (β = 1.069, *p* = 0.136). The BDI, however, showed a pattern by sex that was opposite that of the CVLT, trial 1 score, with a stronger association among women (β = 2.177, *p* = 0.009) than men (β = 1.269, *p* = 0.089). When age was categorized into three groups and examined together with sex, the strongest association was for women 55–60 years of age (β = 1.976, *p* = 0.169).

Finally, multivariate models were constructed for these two tests with individual serum congener concentrations (Table 5). For the CVLT, trial 1 score, significant negative associations were apparent for PCB-105, 118, 138, 170, 180, and 194. For the BDI, the significant positive associations were observed for PCB-153 through 194. A similar multivariate analysis of PCB TEQs revealed that scores on the BDI increased as PCB–TEQ concentration increased (β = 0.266 per unit change in log adjusted lipid basis TEQ, *p* = 0.065, data not shown).

## Discussion

Clinically, patterns of performance on the CVLT are used to detect learning and memory impairments in patients with dementia, brain tumors, and brain injury (Delis et al. 2000). More specifically, the trial 1 score measures the capacity to learn and retain immediate verbal information, an ability that decreased as serum total PCB concentration increased. The stronger association among men is consistent with the fact that women usually outperform men on the CVLT (Ragland et al. 2000). The reason this association was greatest among men 55–60 years of age is unclear. On the one hand, such a finding appears to contradict the notion that older persons are at higher risk of neurotoxicity from PCBs. On the other hand, it may reflect an acceleration of age-related deficits that are normally not observed until 65 years of age (Paolo et al. 1997) in a group that is already at high risk as a result of their sex. The finding that the association was only significant among those whose annual income was < $45,000 is similar to that found in some studies of children that indicate that the effects of low-level exposure to neurotoxins such as lead are greater among those from poorer socioeconomic backgrounds (Bellinger 2004).

The BDI, which measures the occurrence of 21 symptoms of depression, increased as serum total PCB concentrations increased. The association was stronger among women than men, perhaps reflecting the greater risk of depression among women (Culbertson 1997). The sex difference for depression was age dependent, with the magnitude of the association strongest for women 55–60 years of age, again suggesting a greater susceptibility to PCB-related effects in this age group.

The current study was designed to replicate and expand upon the work of Schantz et al. (1999, 2001). In that study, neuropsychological assessments similar to those conducted here were performed on 101 Lake Michigan fish consumers and 78 non-fish-eating controls 49–86 years of age. The results indicated that three tests of learning and memory were negatively associated with PCB exposure. They included the CVLT, trial 1 score and semantic cluster ratio, and the WMS test of logical delayed recall. The first test was also significantly and negatively associated with PCB exposure in the current investigation, whereas the other two tests were not. The discrepancy in results for the latter two tests may be a consequence of differences in current PCB body burden between the two studies. In Schantz et al. (2001), the median serum total PCB concentration was 7.9 ppb (wet weight), with a maximum of 75 ppb. In contrast, the median serum total PCB concentration for the current study was 3.2 ppb, with a maximum of 19.3 ppb. As in the present study, Schantz et al. (2001) found no tests of other cognitive domains or of motor function were associated with PCB exposure.

The current study builds on the work of Schantz et al. (2001) by extending the outcome assessment to include affective state and olfactory function and the exposure assessment to include individual PCB congeners. Regarding the latter issue, the CVLT, trial 1 score and the BDI were associated with multiple serum PCB congeners, particularly the more heavily chlorinated congeners. Similar findings have been reported for neurobehavioral outcomes in PCB-exposed children enrolled in the Oswego Newborn and Infant Development Study (Stewart et al. 2000). These findings contradict animal and *in vitro* research that suggests that lightly chlorinated congeners, especially those with *ortho*-substitution such as PCB-28, have the greatest potential for neurotoxicity (Seegal 2001). This apparent discrepancy may be explained by the fact that the concentrations of the more heavily chlorinated congeners such as PCBs 153, 180, and 138 were 10 times greater than PCB-28 in this and most other human studies (Hansen 1998). In addition, congener concentrations in human samples are highly intercorrelated (Longnecker et al. 2000), making it difficult to identify which congeners are responsible for observed associations.

Schantz et al. (2001) did not examine depression as an outcome, but depression has been linked to PCBs in some studies of occupationally or environmentally exposed adults. For example, complaints of depression, memory loss, insomnia, and nervousness have been reported among workers exposed to PCBs at capacitor plants (Fischbein et al. 1979). More recently, Peper et al. (2005) assessed the neuropsychological status of 30 employees exposed to indoor air contaminated with PCBs from elastic sealants in a German school building compared with that of 30 unexposed controls. The exposed group tended to report more symptoms characteristic of depression, such as distractibility, tiredness, slowness, and less emotional well-being. The mean serum total PCB concentration for the exposed group was 4.5 ppb in the German study, a value more comparable to that of the current study than that of the Lake Michigan fish eaters studied by Schantz et al. (2001).

In addition to the CVLT-trial 1 score, serum total PCB concentrations were significantly associated with a second test of memory and learning, the WMS test of visual immediate recall in the current study. In this case, however, the association was opposite to that hypothesized, with performance increasing as serum total PCB concentrations increased. The reason for this unexpected finding is unclear. Interestingly, the exposed group in the German indoor air study also performed better on the WMS tests of visual immediate and delayed recall than did the controls. Peper et al. (2005) did not include the CVLT, so direct comparison of results for that test is not possible. Schantz et al. (2001) also reported an anomalous finding, with performance on the WMS test of delayed logical memory improving with increasing serum DDE concentration.

Regarding mechanisms, the pathways by which PCBs may affect neuropsychological status in humans are not well understood. As noted previously, alterations in brain levels of neurotransmitters such as dopamine and serotonin may be involved. Dopamine depletion has also been hypothesized as the explanation for excess mortality from Parkinson disease among women who have been occupationally exposed to PCBs in the capacitor industry (Steenland et al. 2006). Another possible mechanism is through the thyroid system. PCB exposure has been related to changes in thyroid hormone levels in humans (Hagmar 2003), and alterations in thyroid hormones have been associated with adult neuropsychological deficits (Wilson et al. 1998).

Caution must be exercised in the interpretation of this study’s results. First, performance on only 1 of the 10 subtests of the CVLT significantly declined with serum PCB concentrations. Schantz et al. (2001) found significant associations with only 2 CVLT subtests despite the higher level of exposure in that study, but positive results with additional subtests certainly would have helped to strengthen confidence in our findings. Second, contrary to expectations, performance on another test of memory and learning, the WMS test of visual immediate recall, significantly improved with serum PCB concentration. The meaning of this finding is unclear, but it is consistent with a similar observation by Peper et al. (2005). Third, multiple statistical comparisons were performed at a nominal error of 5% per test; therefore, it is possible that the observed associations are due to chance. One solution would have been to adjust the *p*-value to account for the 34 tests, but many believe that such an approach may be inappropriate (Savitz and Olshan 1995). Another procedure would have been to combine tests across domains and assess global effects. This method was not followed for two major reasons: *a*) comparability, because Schantz et al. (2001), Peper et al. (2005), and many other investigations have analyzed each test individually; and *b*) interpretability, because even tests within the same domain do not measure exactly the same function.

Instead, we chose to focus on the concordance of our results with those of other studies to help differentiate valid from spurious associations. The fact that the one subtest of the CVLT that was significant in the current study was also one of the two reported by Schantz et al. (2001) helps to validate that finding. In addition, blood concentrations of PCBs were unrelated to any measures of executive function, visual spatial function, or motor function in either study. Other studies also have found that depressive symptoms are more common among PCB-exposed persons. Although unpredicted, the finding of improving performance on the WMS test of visual immediate recall with serum PCB concentration is also consistent with at least one previous study of PCB-exposed adults.

Strengths of the current study include sensitive and objective measures of exposure and outcome, a focus on long-term residence to maximize the opportunity for exposure, and a comprehensive set of potential confounders. The results are consistent with the only other study of PCB exposure and neuropsychological status in older adults, which also found memory and learning deficits in persons with higher serum PCB concentrations. The data are also consistent with other research reporting a higher prevalence of depressive symptoms among PCB-exposed adults. Given the possibility of spurious associations due to multiple statistical comparisons and the contrary-to-expected finding of a protective effect for one test of memory and learning, additional studies of PCB exposure among older persons are needed to determine more definitely whether they are a sensitive subgroup.

## Conclusion

The results suggest that higher serum PCB concentrations are associated with decreased verbal learning as measured by the CVLT trial 1 score and with increased symptoms of depression as measured by the BDI among adults 55–75 years of age. The WMS test of visual immediate recall unexpectedly showed the opposite pattern, with performance increasing as serum total PCB concentrations increased. A similar finding has been reported in a German study of indoor air PCB exposure, and its meaning is unclear. Other cognitive domains, motor function, and olfaction were not associated with PCB exposure. Caution must be exercised in the interpretation of these results. Nevertheless, the study complements the work of Schantz et al. (2001) and suggests that exposures to PCBs may be associated with some measures of memory, learning and depression among adults 55–74 years of age whose current body burdens are similar to those of the general population. Although the results are useful in delineating the neuropsychological effects of low-level exposure to PCBs, further studies of whether older men and women are a sensitive subpopulation are needed.

## Corrections

In the original manuscript published online, there were three errors that have been corrected here:

In the first paragraph of the “Results” (p. 211), the mean serum total PCB concentration has been changed from 520 ppb (lipid basis) to 537 ppb.

In the first and second paragraphs of the “Discussion” (p. 213), the age range has been changed from 50–55 years of age to 55–60 years of age.

## Figures and Tables

**Table 1:** Background characteristics of study participants.

Characteristic	Mean ± SD (range)
Age (years)	63.9 ± 6.0 (55–74)
BMI	28.8 ± 6.3 (16.7–71.3)
Cigarette smoking (packs in last year)	54.0 ± 138.9 (0–730)
Alcohol consumption (drinks per week)	4.4 ± 6.6 (0–42)
Intellectual function (IQ)	107.6 ± 8.8 (88.0–123.1)
Environmental contaminant concentrations	
Serum PCB (ppb)	
Wet weight	3.6 ± 2.0 (1.0–19.3)
Lipid basis	536.8 ± 287.4 (139.3–2116.6)
Serum DDE + DDT (ppb)	
Wet weight	4.0 ± 3.6 (0–22.3)
Lipid basis	738.4 ± 698.3 (3.0–4,082.2)
Serum PCB–TEQ (ppt–lipid basis)	34.2 ± 128.8 (0.0–483)
Blood lead (μg/dL)	3.1 ± 2.0 (0.5–17.7)
Blood mercury (μg/dL)	0.3 ± 0.3 (0.1–2.1)

	No. (%)^a^

Sex (male)	127 (50)
Education	
Less than high school diploma	20 (7.9)
High school diploma	88 (34.8)
Some college	65 (25.7)
4-year college or more	80 (31.6)
Occupational exposures (lifetime)^b^	
Solvents	90 (35.6)
Lead	26 (10.3)

a The total number of responses varies because of missing data.

b Probably exposed, as determined by industrial hygenist.

**Table 2:** List of covariates included in final multivariate models by domain and neuropsychological test.

Domain and test	Covariates (high-risk group)
Memory and learning	
CVLT, t-score^a^	Sex (male), education (−), IQ (−), occupational solvent exposure (yes)
CVLT, trial 1 score^a^	Sex (male), age (+), education (−), IQ (−), smoking (+),activity (−)
CVLT, short-delay free recall^a^	Sex (male), IQ (−), employed (no), blood mercury (+)
CVLT, long-delay free recall^a^	Sex (male), income (< $45,000), IQ (−)
CVLT, semantic cluster ratio^a^	Sex (male), IQ (−), ACE inhibitors (yes), occupational solvent exposure (yes)
CVLT, learning slope^a^	IQ (−), smoking (+), hobby personal solvent exposure^c^ (yes), hobby personal pesticide exposure^c^ (yes), occupational solvent exposure (yes)
CVLT, perseverations^b^	Hobby household pesticide exposure^d^ (yes)
CVLT, discriminability^a^	Sex (male), IQ (−), diuretics (yes), thyroid hormones (yes)
CVLT, proactive interference^b^	No covariates
CVLT, recognition hits vs long-delay free recall^a^	Sex (male), income (< $45,000), IQ (−)
WMS, logical immediate recall^a^	Income (< $45,000), IQ (−), hours of sleep (+)
WMS, logical delayed recall^a^	Income (< $45,000), IQ (−), hours of sleep (+)
WMS, visual immediate recall^a^	Age (+), IQ (−), acetaminophen (yes)
WMS, visual delayed recall^a^	Age (+), IQ (−), trait anxiety (+), acetaminophen (yes)
Executive function	
Trail Making Test-Part A, time to complete^b^	Age (+), education (−), IQ (−), hobby household pesticide exposure^d^ (yes), NSAID (no)
Trail Making Test-Part B, time to complete^b^	Age (+), income (< $45,000), IQ (−)
Stroop Color – Word Test, t-score^a^	Sex (female), education (−), IQ (−), high blood pressure (yes)
WCST, categories completed^a^	Age (+), IQ (−), beta blockers (yes), potassium supplements (no)
WCST, perseverative responses^b^	Age (+), IQ (−)
WCST, perseverative errors^b^	Age (+), IQ (−)
WCST, failures to maintain set^b^	IQ (−), hours of sleep (+), employed (no)
Visual and spatial recognition	
Digit symbol coding^a^	Sex (male), age (+), education (−), IQ (−), smoking (+)
Block design^a^	IQ (−), state anxiety (+)
Reaction time	
Reaction time^b^	Age (+), smoking (+), hours of sleep (−), arthritis (yes), NSAIDs (no)
Motor function	
Static motor steadiness, total time (dominant hand)^b^	Sex (male)
Static motor steadiness, total time (nondominant hand)^b^	No covariates
Pegboard completion time (dominant hand)^b^	Age (+), IQ (−), diabetes (yes), arthritis (yes), anti-depressants (yes)
Pegboard completion time (nondominant hand)^b^	Age (+), IQ (−), smoking (+), arthritis (yes), NSAIDS (no)
Finger tapping (dominant hand)^a^	Sex (female), age (+)
Finger tapping (nondominant hand)^a^	Sex (female)
Affective state	
BDI, total score^b^	BMI (+), trait anxiety (+), employed (yes), gout medications (yes), antidepressants (yes)
Anxiety inventory, state anxiety t-score^b^	BMI (+), Income (< $45,000), trait anxiety (+), activity level (−),sex hormones (yes)
Anxiety Inventory, trait anxiety t-score^b^	Depression (+), state anxiety (+), employed (yes), sex hormones (yes)
Olfactory function	
Smell identification test^a^	Age (+), marital status (not married/no live-in partner), smoking (+)

Abbreviations: −, low score at high risk; +, high score at high risk; ACE, angiotensin-converting enzyme.

a Low score = impairment.

b High score = impairment.

c Hobby personal exposures refers to nonoccupational exposure while personally using the substance.

d Hobby household exposure refers to nonoccupational exposure while someone else in the house is using the substance.

**Table 3:** Final multivariate models^a^ for neuropsychological tests with serum total PCB concentration (lipid basis and log transformed).

Domain and test	*n*	β	SE	*p*-Value
Memory and learning				
CVLT, t-score^b^	240	0.706	1.601	0.660
CVLT, trial 1 score^b^	238	−0.576	0.271	0.035
CVLT, short-delay free recall^b^	235	0.048	0.390	0.901
CVLT, long-delay free recall^b^	237	0.551	0.424	0.195
CVLT, semantic cluster ratio^b^	243	−0.008	0.104	0.938
CVLT, learning slope^b^	242	0.127	0.075	0.090
CVLT, perseverations^c^	245	0.013	0.117	0.913
CVLT, discriminability^b^	243	0.017	0.014	0.238
CVLT, proactive interference^c^	245	0.102	0.112	0.366
CVLT, recognition hits vs long-delay free recall^b^	237	0.392	0.313	0.210
WMS, logical immediate recall^b^	233	0.180	0.628	0.775
WMS, logical delayed recall^b^	233	0.133	0.562	0.813
WMS, visual immediate recall^b^	243	1.016	0.403	0.012
WMS, visual delayed recall^b^	242	0.696	0.430	0.107
Executive function				
Trail Making Test-Part A, time to complete^c^	242	−0.036	0.039	0.351
Trail Making Test-Part B, time to complete^c^	233	0.003	0.047	0.947
Stroop Color – Word Test, t-score^b^	243	0.374	0.977	0.702
WCST, categories completed^b^	245	0.101	0.296	0.734
WCST, perseverative responses^c^	234	0.085	0.107	0.427
WCST, perseverative errors^c^	234	0.073	0.099	0.462
WCST, failures to maintain set^c^	230	0.335	0.296	0.257
Visual and spatial recognition				
Digit symbol coding^b^	242	1.713	1.340	0.202
Block design^b^	243	−0.044	1.153	0.969
Reaction time				
Reaction time^c^	231	−0.003	0.012	0.836
Motor function				
Static motor steadiness, total time (dominant)^c^	244	−0.031	0.084	0.713
Static motor steadiness, total time (nondominant)^c^	242	−0.146	0.131	0.266
Pegboard completion time (dominant)^c^	239	−0.029	0.028	0.298
Pegboard completion time (nondominant)^c^	237	−0.026	0.031	0.401
Finger tapping (dominant)^b^	244	−0.943	0.928	0.310
Finger tapping (nondominant)^b^	243	−0.587	0.765	0.444
Affective state				
BDI, total score^c^	243	1.189	0.438	0.007
Anxiety inventory, state anxiety t-score^c^	233	−1.249	1.037	0.230
Anxiety inventory, trait anxiety t-score^c^	243	−0.380	0.935	0.685
Olfactory function				
Smell identification test^b^	244	−0.055	0.299	0.855

β= change in test score per unit change in log transformed lipid basis serum PCB.

a Adjusted for covariates in Table 2; missing data for *n* < 245.

b Low score = impairment.

c High score = impairment.

**Table 4:** Adjusted^a^ mean (SE) score for CVLT), trial 1 and BDI) by total serum PCB quartile (ppb–lipid basis).

	Quartile				
Domain and test	First (*n* = 63)	Second (*n* = 64)	Third (*n* = 63)	Fourth (*n* = 63)	*p*-Value^b^
Memory and learning					
CVLT, trial 1 score	6.66 (0.40)	6.75 (0.40)	6.67 (0.40)	5.97 (0.40)	0.057
Affective state					
BDI	5.04 (0.24)	4.62 (0.24)	5.48 (0.24)	5.96 (0.25)	0.048

a Adjusted for covariates in Table 2.

b Test for linear trend.

**Table 5:** Final multivariate models^a^ for California Verbal Learning Test (CVLT) trial 1 score and Beck Depression Inventory (BDI) with serum PCB congener concentrations (lipid basis and log transformed).

Test	Congener	β	SE	*p*-Value
CVLT, trial 1 score	PCB-28	0.234	0.154	0.130
	PCB-74	0.259	0.168	0.126
	PCB-99	0.158	0.151	0.297
	PCB-105	−0.365	0.155	0.019
	PCB-118	−0.336	0.154	0.030
	PCB-138	−0.415	0.200	0.039
	PCB-153	−0.336	0.229	0.143
	PCB-170	−0.578	0.255	0.024
	PCB-180	−0.462	0.218	0.035
	PCB-183	−0.349	0.201	0.084
	PCB-187	−0.413	0.249	0.099
	PCB-194	−0.592	0.294	0.046
BDI	PCB-28	0.169	0.250	0.500
	PCB-74	0.301	0.280	0.283
	PCB-99	0.449	0.253	0.077
	PCB-105	0.035	0.251	0.889
	PCB-118	0.182	0.247	0.462
	PCB-138	0.591	0.333	0.078
	PCB-153	0.975	0.378	0.011
	PCB-170	1.383	0.397	0.001
	PCB-180	0.992	0.337	0.004
	PCB-183	0.982	0.318	0.002
	PCB-187	1.372	0.393	0.001
	PCB-194	1.471	0.449	0.001

β= change in test score per unit change in log transformed lipid basis serum PCB.

a Adjusted for covariates in Table 2.
